# Supplementary material for: Identification of photoperiod-induced specific miRNAs in the adrenal glands of Sunite sheep (Ovis aries)
Source: Front Vet Sci. 2022 Jul 22;9:888207. doi: 10.3389/fvets.2022.888207 (PMC9354845; doi:10.3389/fvets.2022.888207)
Supplement: Supplementary file 1 [file Data_Sheet_1.ZIP › Supplementary file/miRDeep2 file.zip!/miRDeep2 file/Know_Structure/SP42A2/oar-mir-376b.pdf]

miRBase precursor : oar-mir-376b
Total read count : 596
oar-miR-376e-5p read count : 259
oar-miR-376b-5p read count : 259
oar-miR-376b-3p read count : 77
remaining reads : 1

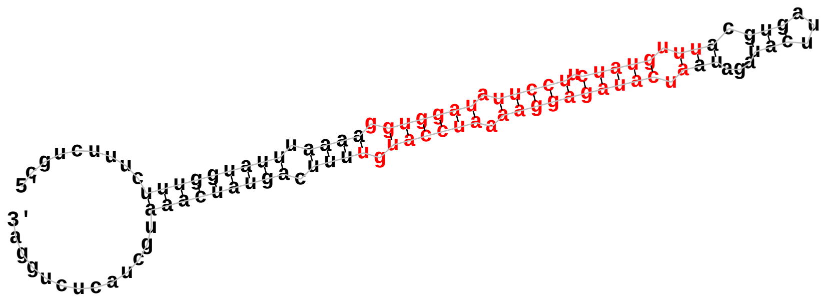

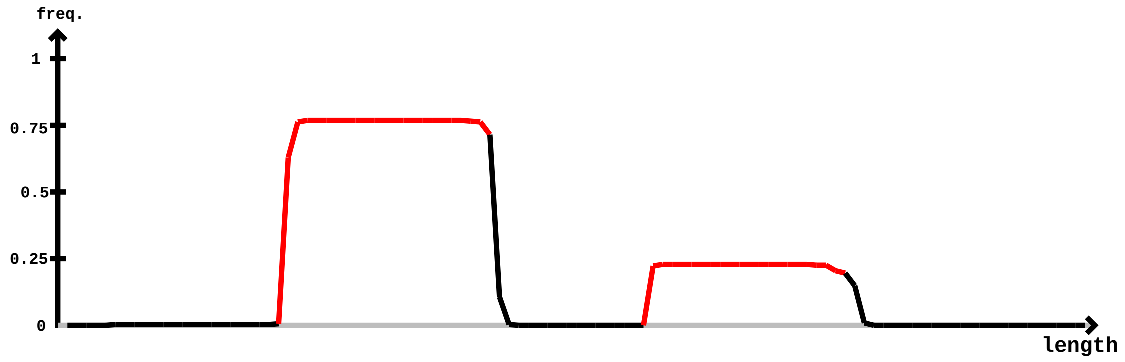

oar-miR-376b-3p

oar-miR-376b-5p

oar-miR-376e-5p

5'- cgucuuucuuugguauuuaaaagguggauauuccuucuauguuuacgugauucauagauaaucauagaggaaaauccauguuuucaguaucaaaugcuacucugga -3' exp

........(((((((((.((((.((((((.(((((.(((((.(((.(((...)))...))).)))))))))).)))))).)))).)))))))))..........	reads	mm	sample
....uuucuuugguauuuaaaag...	1	0	seq
.....................agguggauauuccuucuauguuu..	1	0	seq
......................gguggauauuccuucuaugu..	1	0	seq
......................gguggauauuccuucuauguu...	12	0	seq
......................gguggauauuccuucuauguuu..	172	0	seq
......................gguggauauuccuucuauguuua...	25	0	seq
.......................guggauauuccuucuauguu...	4	0	seq
.......................guggauauuccuucuauguuu..	31	0	seq
.......................guggauauuccuucuauguuua...	10	0	seq
.......................guggauauuccuucuauguuuac..	1	0	seq
........................uggauauuccuucuaug...	1	0	seq
........................uggauauuccuucuauguuu..	1	0	seq
....aucauagaggaaaaucc.............................	1	0	seq
....aucauagaggaaaauccau...........................	7	0	seq
....aucauagaggaaaauccaug..........................	3	0	seq
....aucauagaggaaaauccaugu.........................	15	0	seq
....aucauagaggaaaauccauguu........................	47	0	seq
....aucauagaggaaaauccauguuu.......................	2	0	seq
....ucauagaggaaaauccaugu.........................	1	0	seq
....ucauagaggaaaauccauguuu.......................	1	0	seq
